# Fluorescence from pentacyanopropenide in melamine†

**DOI:** 10.1039/d5ma00400d

**Published:** 2025-07-03

**Authors:** Hanen Mechi, Arthur Mantel, Vipin Mishra, Yuto Urano, Ryo Kitaura, Hidetsugu Shiozawa

**Affiliations:** a J. Heyrovsky Institute of Physical Chemistry, Czech Academy of Sciences Dolejskova 3, 182 23 Prague 8 Czech Republic hide.shiozawa@jh-inst.cas.cz; b Research Center for Materials Nanoarchitectonics, National Institute for Materials Science 1-1 Namiki Tsukuba 305-0044 Japan; c Graduate School of Chemical Science and Engineering, Hokkaido University Kita13, Nishi 8, Kita-ku Sapporo 060-8628 Japan; d Faculty of Physics, University of Vienna Boltzmanngasse 5 1090 Vienna Austria hidetsugu.shiozawa@univie.ac.at

## Abstract

Aggregation-induced optical phenomena are at the forefront of modern materials science. In this work, tetracyanoethylene (TCNE) is reacted and encapsulated within melamine. Crystallization from aqueous tetrahydrofuran solutions containing melamine and TCNE at varying concentrations yields colorful crystals exhibiting multi-wavelength fluorescence emission. Combined infrared spectroscopy and mass spectrometry reveal that the crystals are melamine doped with trace amounts of 1,1,2,3,3-pentacyanopropenide. Fluorescence excitation–emission spectral mapping elucidates the concentration dependence of fluorescence emission in both the precursor solutions and the resulting crystals. Density functional theory calculations attribute the observed multi-wavelength emission to dimers of the pentacyanopropenide. Encapsulating reactive molecules within crystalline melamine, as demonstrated with 1,1,2,3,3-pentacyanopropenide and its dimer, offers a versatile strategy for stabilizing a wide range of otherwise unstable species.

## Abstract 2

A dimer of pentacyanopropenide encapsulated in a single crystal of melamine exhibits strong fluorescence emission in the visible range.

## Graphical abstract

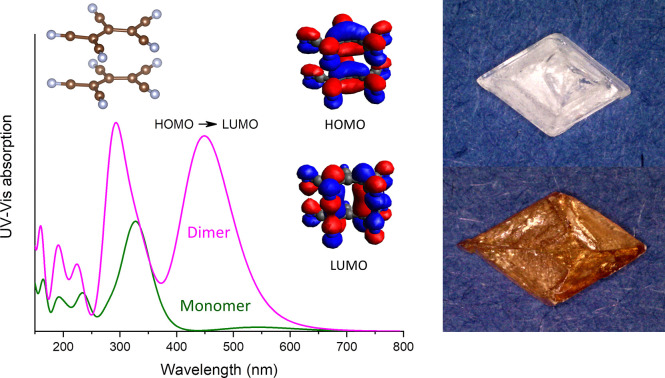

## 1 Introduction

Concentration-dependent luminescence is a fascinating phenomenon in which the intensity or color of emission changes as the concentration of a luminophore varies. The exact behavior depends on various factors, including the properties of the luminophore, environmental conditions, and whether the sample is a solid or a solution. In several important applications, such as organic light-emitting diodes (OLEDs),^1^ bioimaging^2^ and chemical sensing,^3^ the relationship between concentration and luminescent properties is a critical factor to consider.

In many aggregates or solids of fluorophores, fluorescence tends to be quenched.^4–6^ The optical properties of monomers, dimers and trimers in solution have been studied extensively for known fluorophores, such as rhodamine G,^7–11^ which revealed lowered quantum efficiencies for aggregates. Isolated fluorophores in solids^12^ or diluted solutions^6^ can exhibit enhanced emission. Conversely, there are cases where fluorescence is enhanced or even induced by aggregation.^6,13^

In this work, tetracyanoethylene (TCNE) is reacted in a 1 : 1 (v/v) mixture of THF and water to yield 1,1,2,3,3-pentacyanopropenide, which is subsequently encapsulated within melamine crystals. Due to the strong electron affinity of the pentacyano group, 1,1,2,3,3-pentacyanopropenide is stable under ambient conditions only when paired with a cation in salts.^14^ As a result, the optical properties of its neutral or isolated form have not been accessible for study. Melamine (2,4,6-triamino-1,3,5-triazine) has been chosen as the host matrix due to its ability to incorporate dopants effectively.^15^ Being colorless and absorbing only in the ultraviolet region, melamine is transparent in the visible range and exhibits fluorescence only in the ultraviolet region, making it an ideal host material for encapsulating reactive species and studying their optical properties.

We observed that colorful 1,1,2,3,3-pentacyanopropenide-doped melamine crystals exhibit fluorescence across multiple wavelengths in the visible spectrum. The emission wavelength increases systematically with the concentration of 1,1,2,3,3-pentacyanopropenide. Density functional theory (DFT) calculations suggest that this concentration-dependent fluorescence behavior arises from the formation of pentacyanopropenide aggregates within the crystal structure.

## 2 Results and discussion

### 2.1 Synthesis

**Fig. 1:**
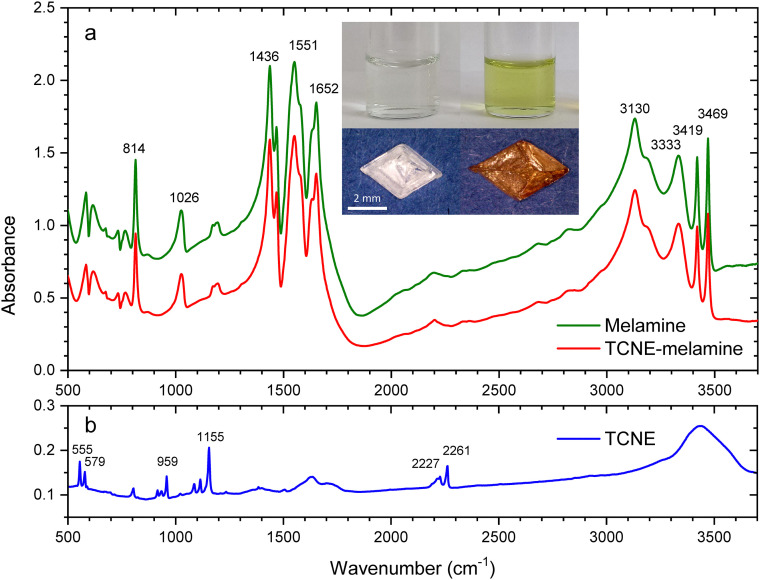
(a) Infrared absorption spectra of pure melamine and doped melamine crystals. Insets: Micrographs of crystals (bottom left and bottom right) crystallized from precursor solutions prepared by mixing 1 mL of a 100 mM aqueous solution of melamine with 1 mL of THF solutions of TCNE at concentrations of 0.2 mM (top left) and 20 mM (top right). (b) Infrared spectrum of TCNE crystals. The samples were finely ground and blended with spectroscopic-grade KBr in a ratio of approximately 1 : 100 (sample : KBr) before being compressed into pellets.

Doped melamine crystals were synthesized *via* crystallization of melamine in a 1 : 1 (v/v) mixture of deionized water and THF, with varying concentrations of TCNE. For further details, see the ESI.† The inset of Fig. 1 shows micrographs of crystals prepared by mixing 1 mL of a 100 mM aqueous solution of melamine with 1 mL of THF solutions of TCNE at concentrations of 0.2 mM (bottom left) and 20 mM (bottom right).

### 2.2 Infrared spectroscopy

Fig. 1 displays the infrared absorption spectra of melamine, TCNE, and doped melamine crystals. The spectrum of melamine exhibits characteristic modes of the triazine ring at 814, 1026, 1436 and 1551 cm^−1^, a bending mode of –NH_2_ at 1652 cm^−1^, and stretching modes of –NH_2_ at 3130, 3333, 3419 and 3469 cm^−1^.^16^ The spectrum of TCNE shows peaks at 555 and 579 cm^−1^, corresponding to stretching vibrations of the C=C–C group.^17^ Peaks at 959 and 1155 cm^−1^ are attributed to the stretching vibrations of the C=C bond, while those at 2227 and 2261 cm^−1^ correspond to the stretching vibrations of the CN bond in the cyano group. Importantly, the spectrum of the doped melamine retains all the peaks of pure melamine, but no peaks from TCNE, suggesting that the color of the crystals arises from impurity species embedded within the melamine structure.

### 2.3 Mass spectrometry

**Fig. 2:**
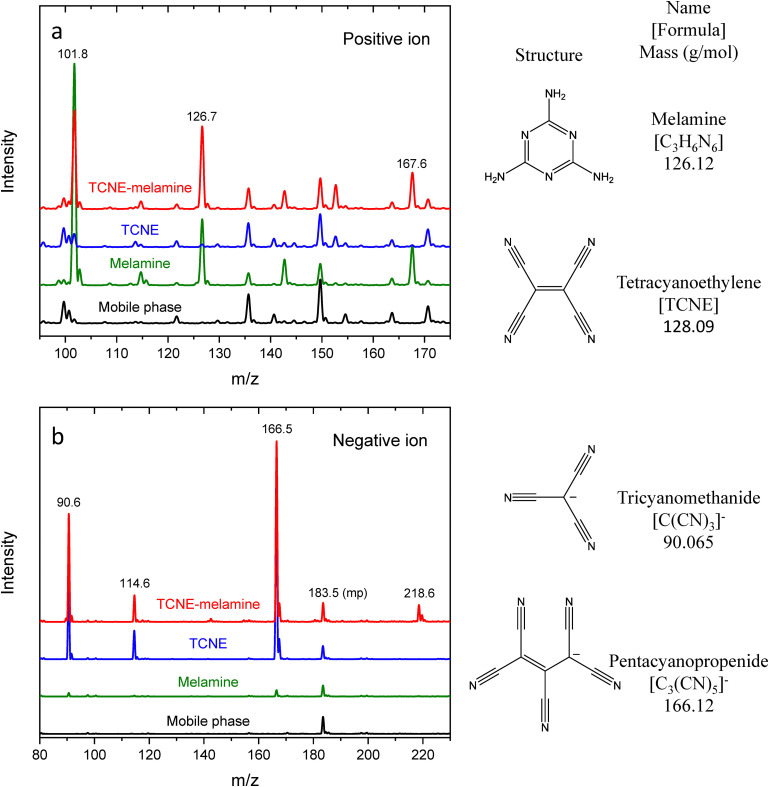
The mass spectra of a THF solution of TCNE, an aqueous solution of melamine, and a 1 : 1 (v/v) aqueous THF solution of TCNE and melamine (TCNE–melamine), measured in the (a) positive ion mode and (b) negative ion mode.

Mass spectrometry is a powerful analytical tool used to obtain information about the chemical structure and composition of compounds. Fig. 2 presents the mass spectra of a THF solution of TCNE, an aqueous solution of melamine, and a 1 : 1 (v/v) aqueous THF solution of TCNE and melamine (TCNE–melamine), measured in both the positive ion mode (panel (a)) and the negative ion mode (panel (b)). In the positive ion spectra of all samples, multiple mobile phase peaks are observed. The intense peaks at *m*/*z* = 42.04 and 82.67 correspond to the protonated acetonitrile [ACN + H]^+^ (molecular mass of 42.06 g mol^−1^) and its dimer [2ACN + H]^+^ (83.11 g mol^−1^), respectively. Additionally, the mass spectra of melamine and TCNE–melamine display distinct peaks at *m*/*z* = 101.8, 126.7 and 167.6. The peak at *m*/*z* = 126.92 corresponds to the [melamine + H]^+^ ion (molecular mass of 127.13 g mol^−1^),^18,19^ while *m*/*z* = 167.6 is attributed to an adduct of melamine and acetonitrile, [melamine + ACN + H]^+^ (168.18 g mol^−1^).^20^*m*/*z* = 101.8 may correspond to a decomposition product of melamine.

In the negative ion mode, three distinct peaks are observed at *m*/*z* = 90.6, 114.6 and 166.5 in the spectra of both TCNE and TCNE–melamine. The negative ion mode generates peaks for molecules with a higher electron affinity or those that readily accept electrons. No peaks corresponding to the radical anion [TCNE]˙^−^ (molecular mass of 128.09 g mol^−1^) are observed. The largest peak at *m*/*z* = 166.5 can be attributed to 1,1,2,3,3-pentacyanopropenide [C_3_(CN)_5_]^−^ (molecular mass of 166.12 g mol^−1^).^20^ The molecular structure is illustrated in the bottom right of Fig. 2. The peak at *m*/*z* = 114.6 indicates the formation of [C_3_(CN)_3_]^−^ (molecular mass of 114.087 g mol^−1^).^21^ The peak at *m*/*z* = 90.6 is attributed to tricyanomethanide [C(CN)_3_]^−^ (molecular mass of 90.065 g mol^−1^).^20,22^ The peak at *m*/*z* = 218.6, observed only in TCNE–melamine, suggests the presence of an adduct anion of TCNE products and melamine, such as [C_3_(CN)_5_ + 2(CN)]^−^.

**Fig. 3:**
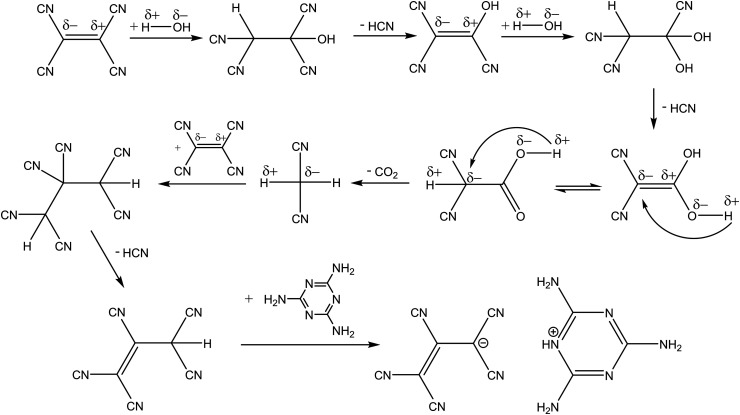
Chemical equation illustrating the formation of 1,1,2,3,3,-pentacyanopropenide in aqueous melamine.

It has been reported that TCNE reacts in water to form 1,1,2,3,3-pentacyanopropenide [C_3_(CN)_5_]^−^ (molecular mass of 166.12 g mol^−1^) under basic conditions^20,23^ and tricyanoethanolate [C_2_(CN)_3_O]^−^ (molecular mass of 118.075 g mol^−1^) under neutral and acidic conditions.^24^ In our case, aqueous melamine acts as a weak base. Fig. 3 shows a possible reaction pathway leading to the formation of 1,1,2,3,3-pentacyanopropenide.

**Fig. 4:**
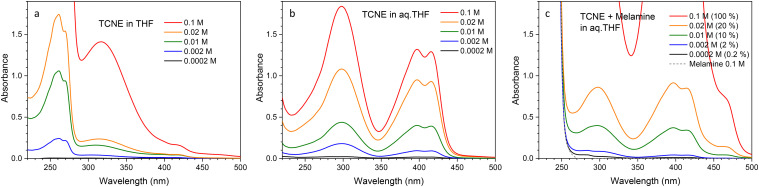
(a) UV-Vis spectra of aqueous solutions of TCNE at concentrations of 0.2, 2.0, 10 and 20 mM. (b) UV-Vis spectra of aqueous THF solutions of TCNE at concentrations of 0.2, 2.0, 10 and 20 mM. (c) UV-Vis spectra of precursor solutions prepared by mixing a 100 mM aqueous solution of melamine and a THF solution of TCNE at concentrations of 0.2 mM (0.2%), 2.0 mM (2.0%), 10 mM (10%) and 20 mM (20%), where the percentages in parentheses represent the molar concentration ratios of TCNE to melamine.

It should be noted that the observed ion species could also be generated *via* electron ionization. However, mass spectrometry using inert buffer gas detected [TCNE]˙^−^ (molecular mass of 128.09 g mol^−1^) as the major adduct anion,^21,25^ with no evidence of 1,1,2,3,3-pentacyanopropenide [C_3_(CN)_5_]^−^ (molecular mass of 166.12 g mol^−1^). This suggests that 1,1,2,3,3-pentacyanopropenide was present in the measured THF solution of TCNE and the 1 : 1 (v/v) aqueous THF solution of TCNE and melamine. Since water is part of the mobile phase in the HPLC–MS system, TCNE should have reacted with water to yield 1,1,2,3,3-pentacyanopropenide. Therefore, the main UV-Vis absorption features observed for both the aqueous THF solution of TCNE and the aqueous THF solution of TCNE and melamine (Fig. 4b and c) should correspond to 1,1,2,3,3-pentacyanopropenide. Hence, 1,1,2,3,3-pentacyanopropenide is likely the species embedded in the melamine crystals.

### 2.4 Ultraviolet-visible absorption spectroscopy

Fig. 4 shows the UV-Vis absorption spectra of aqueous solutions of TCNE at concentrations of 0.2, 2.0, 10, 20 and 100 mM (panel (a)), 1 : 1 (v/v) aqueous THF solutions of TCNE at the same concentrations (panel (b)), and precursor solutions prepared by mixing a 100 mM aqueous solution of melamine with THF solutions of TCNE at concentrations of 0.2, 2.0, 10, 20 and 100 mM (panel (c)). For each solutions, the spectral shape remains unchanged with varying concentrations. The spectra of the THF solutions of TCNE exhibit intense peaks in the UV range, with a sharp upper-wavelength edge at 280 nm. Less intense absorption peaks are observed at longer wavelengths.

According to mass spectrometry, TCNE reacts with water to yield 1,1,2,3,3-pentacyanopropenide in a 1 : 1 (v/v) mixture of THF and water. As a result, the UV-Vis absorption spectra of aqueous TCNE solutions differ significantly from those of TCNE in pure THF. They display two distinct absorption features, as shown in Fig. 4b. The first peak appears at approximately 298 nm. The second feature is a band consisting of multiple absorption lines, located within the wavelength range of 350–450 nm. Similar absorption lines have been reported for a 1,1,2,3,3-pentacyanopropenide salt.^23^

Finally, Fig. 4c shows the UV-Vis absorption spectra of TCNE and melamine dissolved in a 1 : 1 (v/v) mixture of THF and water (the precursor solutions for the doped melamine crystals, in which TCNE is fully converted into 1,1,2,3,3-pentacyanopropenide according to mass spectrometry). These spectra exhibit the UV absorption edge of melamine around 250 nm and a weak absorption near 470 nm, in addition to the peaks observed for pure TCNE in the THF–water mixture. As the absorption at 470 nm is not observed for pure melamine or TCNE in aqueous THF (as shown in panel (b)), it is likely due to an interaction between 1,1,2,3,3-pentacyanopropenide and melamine or a change in the state of 1,1,2,3,3-pentacyanopropenide induced by the presence of melamine, which acts as a weak base in the solution.

### 2.5 Fluorescence

**Fig. 5:**
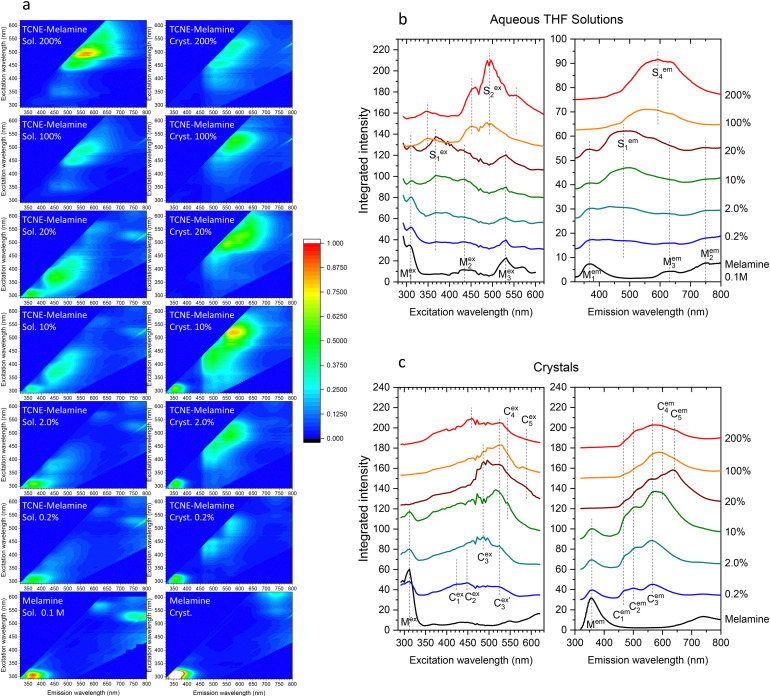
(a) Fluorescence excitation–emission wavelength maps for the precursor aqueous THF solutions of TCNE and melamine (left panels) and the corresponding single crystals (right panels). The solutions were prepared by mixing a 100 mM aqueous solution of melamine with THF solutions of TCNE at molar concentrations of 0.2 mM (0.2%), 2.0 mM (2.0%), 10 mM (10%), 20 mM (20%), 100 mM (100%), and 200 mM (200%), where the values in parentheses indicate the molar percentage of TCNE relative to melamine. Maps for a 100 mM aqueous THF solution of pure melamine and for a pure solid melamine crystal are shown at the bottom for reference. (b) Integrated excitation and emission wavelength profiles for the precursor aqueous THF solutions. (c) Integrated excitation and emission wavelength profiles for the corresponding single crystals.

The doped melamine crystals are not only colorful but also fluorescent. In this section, we present a comprehensive analysis of the fluorescence properties of individual crystals by mapping luminescence intensity as a function of both excitation wavelength (*λ*_ex_) and emission wavelength (*λ*_em_) across the UV-visible spectrum.^26^ The left panels in Fig. 5a show fluorescence excitation–emission maps for aqueous THF solutions of TCNE and melamine. These were prepared by mixing a 100 mM aqueous solution of melamine with THF solutions of TCNE at molar concentrations of 0.2 mM (0.2%), 2.0 mM (2.0%), 10 mM (10%), 20 mM (20%), 100 mM (100%), and 200 mM (200%), where the values in parentheses indicate the molar percentage of TCNE relative to melamine. For comparison, the map for a 100 mM aqueous THF solution of pure melamine is also shown at the bottom.

The corresponding integrated excitation and emission profiles are shown in Fig. 5b. The pure melamine solution exhibits a strong fluorescence emission at *λ*_ex_ = 310 nm (*M*^ex^_1_) and *λ*_em_ = 368 nm (*M*^em^_1_). As the relative TCNE concentration increases from 0 to 20%, the *M*_1_ emission of melamine gradually weakens, while a new emission feature emerges at *λ*_ex_ = 368 nm (*S*^ex^_1_) and *λ*_em_ = 480 nm (*S*^em^_1_). At concentrations above 100%, both the *M*_1_ emission of melamine and the *S*_1_ emission vanish, and multiple new peaks centered around *λ*_ex_ = 493 nm (*S*^ex^_2_) and *λ*_em_ = 593 nm (*S*^em^_2_) appear. Note that subscript indices have been assigned so that the corresponding excitation–emission peak pairs share the same number.

The right panels in Fig. 5a present the fluorescence excitation–emission wavelength maps of single crystals prepared from the respective precursor solutions. Their integrated excitation and emission profiles are shown in Fig. 5c. The *M*_1_ emission of a pure melamine crystal appears at *λ*_ex_ = 312 nm (*M*^ex^_1_) and *λ*_em_ = 358 nm (*M*^em^_1_), which diminishes with increasing TCNE concentration and disappears entirely at 20%. Doped melamine crystals display multiple emission features, with distinct patterns depending on the TCNE concentration. At concentrations of 0.2% and 2.0%, three emission peaks labeled *C*^em^_1_, *C*^em^_2_, and *C*^em^_3_ are observed. At higher concentrations, additional emissions labeled *C*^em^_4_ and *C*^em^_5_ emerge at longer wavelengths, while the *C*^em^_1_, *C*^em^_2_, and *C*^em^_3_ emission peaks remain detectable even at the highest concentration studied.

### 2.6 Fluorescence lifetime

**Fig. 6:**
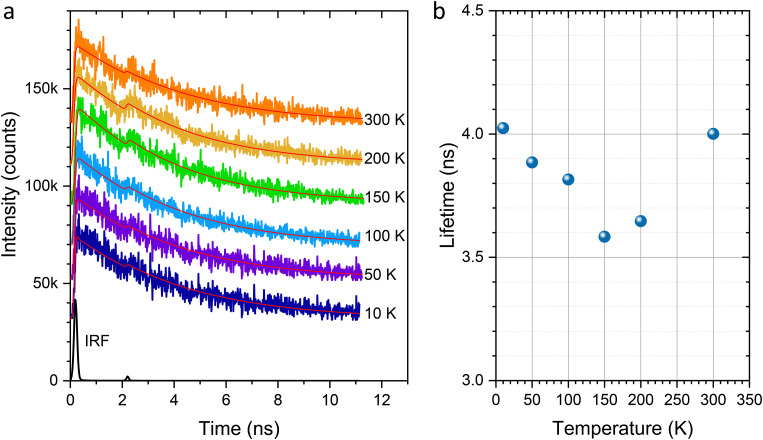
(a) Fluorescence decay profiles measured at an excitation wavelength of 490 nm at various temperatures. (b) Corresponding fluorescence lifetimes as a function of temperature.

Fig. 6a shows the fluorescence decay curves measured for the 2.0% doped crystal at an excitation laser wavelength of 490 nm at various temperatures ranging from 10 to 300 K. The instrument response function (IRF) measured at 10 K is shown in black for reference. The decay curves are well described by a convolution of the IRF with a single-exponential decay function. For details on the fitting analysis, refer to the ESI.† The extracted lifetimes are 4.02, 3.88, 3.82, 3.58, 3.65, and 4.00 ns at 10, 50, 100, 150, 200, and 300 K, respectively, as plotted in Fig. 6b. No clear systematic dependence on temperature is observed, and the average lifetime is 3.83 ns.

### 2.7 Density-functional theory calculations

**Fig. 7:**
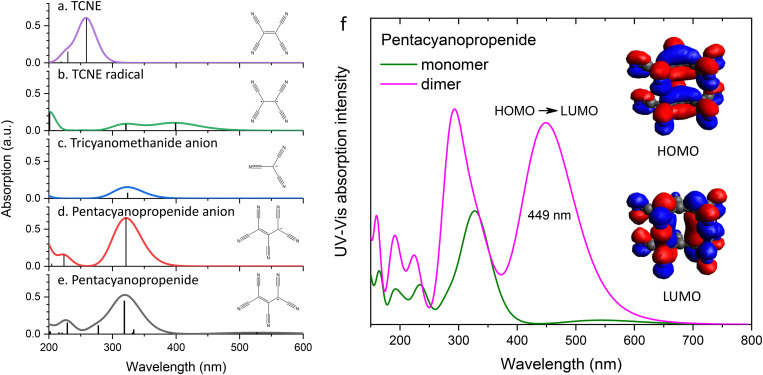
UV-Vis absorption spectra simulated for aqueous solutions of (a) TCNE, (b) the neutral TCNE radical, (c) the tricyanomethanide anion, (d) the pentacyanopropenide anion, and (e) neutral pentacyanopropenide. (f) Simulated UV-Vis spectrum of the 1,1,2,3,3-pentacyanopropenide radical dimer compared with that of the monomer. Insets show the highest occupied molecular orbital (HOMO) and lowest unoccupied molecular orbital (LUMO).

Fig. 7 presents the UV-Vis spectra simulated using density-functional theory calculations. Based on mass spectrometry data, the major species present in the precursor solutions are identified as 1,1,2,3,3-pentacyanopropenide (panel (e)) and tricyanomethanide anions (panel (c)). The calculated UV-Vis spectra for both anions exhibit their most intense absorption peaks in the wavelength range of 325–330 nm, which can be correlated with the experimental band observed at 298 nm in Fig. 4b and c. Notably, neither species shows any strong absorption bands at longer wavelengths. In contrast, the neutral TCNE radical (panel (b)) features a prominent absorption band at 400 nm, but this species is absent in the precursor solutions, as confirmed by the mass spectrometry results shown in Fig. 2.

To account for the experimentally observed absorption features in the wavelength range from 350 to 450 nm, additional DFT calculations were performed on a dimer of 1,1,2,3,3-pentacyanopropenide. Fig. 7f compares the calculated absorption spectrum of the dimer with that of the monomer. The dimer displays a strong absorption band at 449 nm, corresponding to the HOMO–LUMO transition, which matches well with the absorption band observed in the range between 350 and 450 nm in the experimental UV-Vis spectrum (Fig. 4b and c), the *S*^ex^_2_ peak in the excitation wavelength profiles for the 100% and 200% aqueous THF solutions (Fig. 5b), and the *C*^ex^_3_ and 
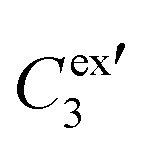
 peaks in the excitation wavelength profiles for the 2–100% crystals (Fig. 5c). These results strongly support the conclusion that the 1,1,2,3,3-pentacyanopropenide dimer is the primary chromophore responsible for the color observed in the doped melamine crystals.

## 3 Conclusions

1,1,2,3,3-Pentacyanopropenide was synthesized and encapsulated in solid melamine at different concentrations. The long-wavelength absorption and emission observed at higher concentrations were attributed to the pentacyanopropenide dimer. Encapsulated in melamine, 1,1,2,3,3-pentacyanopropenide becomes highly stable, which serves as a means to tailor multifunctional optical materials.

## Author contributions

H. M. performed the synthesis, UV-Vis absorption, and fluorescence spectroscopy and wrote the manuscript. A. M. conducted the synthesis, fluorescence spectroscopy, and DFT calculations. V. M. carried out the synthesis. Y. U. and R. K. performed fluorescence spectroscopy and lifetime measurements at various temperatures. H. S. conceptualized and supervised the project, provided resources, carried out data curation and DFT calculations, and wrote the manuscript.

## Conflicts of interest

There are no conflicts of interest to declare.

## Supplementary Material

MA-006-D5MA00400D-s001

## Data Availability

The data supporting this article have been included as part of the ESI.†

## References

[cit1] Burroughes J. H., Bradley D. D. C., Brown A. R., Marks R. N., Mackay K., Friend R. H., Burns P. L., Holmes A. B. (1990). Light-emitting diodes based on conjugated polymers. Nature.

[cit2] Yang Y., Zhao Q., Feng W., Li F. (2013). Luminescent chemodosimeters for bioimaging. Chem. Rev..

[cit3] Zhang B., Ge C., Yao J., Liu Y., Xie H., Fang J. (2015). Selective selenol fluorescent probes: design, synthesis, structural determinants, and biological applications. J. Am. Chem. Soc..

[cit4] Green A. P., Buckley A. R. (2015). Solid state concentration quenching of organic fluorophores in pmma. Phys. Chem. Chem. Phys..

[cit5] Srujana P., Sudhakar P., Radhakrishnan T. P. (2018). Enhancement of fluorescence efficiency from molecules to materials and the critical role of molecular assembly. J. Mater. Chem. C.

[cit6] Han T., Yan D., Wu Q., Song N., Zhang H., Wang D. (2021). Aggregation-induced emission: A rising star in chemistry and materials science. Chin. J. Chem..

[cit7] Arbeloa F. L., Ojeda P. R., Arbeloa I. L. (1988). Dimerization and trimerization of rhodamine 6g in aqueous solution. effect on the fluorescence quantum yield. J. Chem. Soc., Faraday Trans. 2.

[cit8] Taguchi T., Hirayama S., Okamoto M. (1994). New spectroscopic evidence for molecular aggregates of rhodamine 6g in aqueous solution at high pressure. Chem. Phys. Lett..

[cit9] Bojarski P., Matczuk A., Bojarski C., Kawski A., Kukliński B., Zurkowska G., Diehl H. (1996). Fluorescent dimers of rhodamine 6g in concentrated ethylene glycol solution. Chem. Phys..

[cit10] Li R., Fan Y., Tang B., Ren J., Zhang L. (2011). Concentration-dependent luminescent behaviour of rhodamine 6g in alpo4 xerogel monoliths. Mater. Chem. Phys..

[cit11] Barzan M., Hajiesmaeilbaigi F. (2018). Investigation the concentration effect on the absorption and fluorescence properties of rhodamine 6g dye. Optik.

[cit12] Li J., Yuan S., Qin J.-S., Huang L., Bose R., Pang J., Zhang P., Xiao Z., Tan K., Malko A. V. (2020). *et al.*, Fluorescence enhancement in the solid state by isolating perylene fluorophores in metal-organic frameworks. ACS Appl. Mater. Interfaces.

[cit13] Luo J., Xie Z., Lam J. W. Y., Cheng L., Chen H., Qiu C., Kwok H. S., Zhan X., Liu Y., Zhu D. (2001). *et al.*, Aggregation-induced emission of 1-methyl-1, 2, 3, 4, 5-pentaphenylsilole. Chem. Commun..

[cit14] Johnson T. J., Hipps K. W., Willett R. D. (1988). Salts of the 1,1,2,3,3,-pentacyanopropenide anion: crystallographic and spectroscopic studies. J. Phys. Chem..

[cit15] Mishra V., Mantel A., Kapusta P., Prado-Roller A., Shiozawa H. (2024). Highly luminescent tcnq in melamine. ACS Appl. Opt. Mater..

[cit16] Jeremy Jones W., Orville-Thomas W. J. (1959). The infra-red spectrum and structure of melamine. Trans. Faraday Soc..

[cit17] Takenaka T., Tadokoro S.-I., Uyeda N. (1971). Infrared absorption spectra of tetracyanoethylene: Adsorbed on evaporated alkali halides. Bull. Inst. Chem. Res., Kyoto Univ..

[cit18] Yang S., Ding J., Zheng J., Hu B., Li J., Chen H., Zhou Z., Qiao X. (2009). Detection of melamine in milk products by surface desorption atmospheric pressure chemical ionization mass spectrometry. Anal. Chem..

[cit19] Kailasa S. K., Wu H.-F. (2015). Electrospray ionization tandem mass spectrometry for rapid, sensitive and direct detection of melamine in dairy products. J. Ind. Eng. Chem..

[cit20] Miller J. S. (2006). Tetracyanoethylene (tcne): The characteristic geometries and vibrational absorptions of its numerous structures. Angew. Chem., Int. Ed..

[cit21] Smith-Gicklhorn A. M., Frankowski M., Bondybey V. E. (2002). Tetracyanoethylene, its ions and ionic fragments. Phys. Chem. Chem. Phys..

[cit22] Soltner T., Häusler J., Kornath A. J. (2015). The existence of tricyanomethane. Angew. Chem., Int. Ed..

[cit23] Middleton W. J., Little E. L., Coffman D. D., Engelhardt V. A. (1958). Cyanocarbon chemistry. v.1 cyanocarbon acids and their salts. J. Am. Chem. Soc..

[cit24] Conan F., Gall B. L., Kerbaol J.-M., Stang S. L., Sala-Pala J., Mest Y. L., Bacsa J., Ouyang X., Dunbar K. R., Campana. C. F. (2004). Electrochemical, spectroscopic, and structural evidence for the mild hydrolysis of tetracyanoethylene, tcne, to form the 2,3,3-tricyanoacrylamidate ligand: Isolation of an unexpected quadruply-bonded polymeric material [mo_2_(o_2_ccme_3_)_3_((nc)_2_cc(cn)conh)]. Inorg. Chem..

[cit25] Culbertson J. A., Sears L. J., Knighton W. B., Grimsrud E. P. (1992). Origin of adduct ions in the electron-capture mass spectrum of tetracyanoethylene. Org. Mass Spectrom..

[cit26] Guerraf A. E., Zeng W., Mantel A., Benhsina E., Chin J. M., Shiozawa H. (2024). Synchronous electrochromism and electrofluorochromism in a zirconium pyrenetetrabenzoate metal-organic framework. Adv. Electron. Mater..

