# Supplementary material for: Fluorescence from pentacyanopropenide in melamine
Source: Mater Adv. 2025 Jul 3;6(17):5884–91. doi: 10.1039/d5ma00400d (PMC12268318; doi:10.1039/d5ma00400d)
Supplement: MA-006-D5MA00400D-s001 [file MA-006-D5MA00400D-s001.pdf]

Supplementary Information (SI) for Materials Advances.
This journal is © The Royal Society of Chemistry 2025

Supplementary information

Fluorescence from pentacyanopropenide in melamine

Hanen Mechi[1], **Arthur Mantel**[1], **Vipin Mishra**[1], **Yuto Urano**[2,3], **Ryo Kitaura**[2,3], **and Hidetsugu Shiozawa**[1,4*]

[1]J. Heyrovsky Institute of Physical Chemistry, Czech Academy of Sciences, Dolejskova 3, 182 23 Prague 8, Czech Republic

[2]Research Center for Materials Nanoarchitectonics, National Institute for Materials Science, 1-1 Namiki, Tsukuba 305-0044, Japan

[3]Graduate School of Chemical Science and Engineering, Hokkaido University, Kita13, Nishi 8, Kita-ku, Sapporo 060-8628, Japan

[4]Faculty of Physics, University of Vienna, Boltzmanngasse 5, 1090 Vienna, Austria

[*]To whom correspondence should be addressed;

E-mail: hide.shiozawa@jh-inst.cas.cz & hidetsugu.shiozawa@univie.ac.at

S1 Materials

All commercially available chemicals, including TCNE (Sigma-Aldrich) and melamine (Sigma-Aldrich), were used as received from their suppliers. For solvents, tetrahydrofuran (Sigma-Aldrich) and deionized water were used.

S2 Synthesis of TCNE doped melamine Crystals

The doped melamine crystals were prepared as follows. TCNE was dissolved in THF (1 mL) at molar concentrations of 0.2, 0.1, 0.05, 0.02, 0.01, 0.005, 0.002, 0.001, and 0.0002 M at room temperature. A 0.1 M aqueous solution of melamine was prepared by heating melamine (0.0126 g, 0.1 mmol) in water (1 mL) in a glass vial on a hot plave set to 100 °C. After cooling to lukewarm temperature, the melamine solution was mixed with the THF solution of TCNE and stirred for 5 minutes. The resulting mixture was left to evaporate at room temperature overnight, leading to the formation of colored crystals in the residual solvent. The crystals were collected and thoroughly rinsed with methanol several times

S3 Optical microscopy

Optical micrographs of the crystals were acquired using an optical microscope (BX41M-LED, Olympus, Japan) equipped with an Infinity 1 camera (Lumenera, Canada) and QuickPHOTO CAMERA 3.1 software.

S4 HPLC and mass spectroscopy

The high performance liquid chromatography - mass spectroscopy (HPLC-MS) setup combines an Agilent 1260 Infinity HPLC system equipped with dual wavelength detection (256 and 356 nm) in a flow cell of 10 mm path length, and an Advion mass spectrometer (expression CMS-L) to record the mass spectra for both positive and negative ions generated by electrospray ionization (ESI). The Agilent C18 reversed-phase column (Poroshell 120) was used. The HPLC column was operated with a mobile phase composed of a mixture of acetonitrile and water. HPLC-MS experiments were conducted using mixtures of water and acetonitrile as mobile phase with sequential water-to-acetonitrile volume ratios of 95:5 (0.5 min.), 5:95 (22.5 min.) and 50:50 (12.5 min.). Diluted solutions of melamine in water, TCNE in THF, and a mixture of TCNE and melamine (1:1 molar ratio) in aqueous THF (1:1 volume ratio) were measured.

S5 IR spectroscopy

FTIR spectra were recorded using a Nexus 670 E.S.P. spectrometer (Thermo Nicolet Co.). The samples were finely ground and mixed with spectroscopic-grade KBr in a ratio of approximately 1:100 (sample:KBr) before

being compressed into pellets. The spectra were acquired in the range of 4000–400 cm^{-1} and the background correction was performed using a blank KBr pellet.

S6 UV-Vis spectroscopy

UV-Vis spectra were measured on a crystak in a 2mm thick cuvette using Shimazu UV-2600i.

S7 Fluorescence spectroscopy

Fluorescence excitation and emission wavelength maps were measured on a crystal in a quartz cuvette (Ossila, C2003P1) using Horiba Horiba Fluorolog®-3 equipped with a 450 W ozone-free xenon short-arc and R928P photomultiplier tube with DM302 PC Acquisition Module. For both excitation and emission monochrometers, diffraction gratings with a line density of 1200 l/mm and a blaze wavelength of 500 nm were used, and the entrance and exit slits were set to bandpaths of 2, 3 or 4 nm, depending on the fluorescence intensity.

S8 Density functional theory

All density functional theory (DFT) calculations were performed using the ORCA 5.0.4 quantum chemistry package executed on the Ubuntu 22.04.2 LTS operating system in parallel mode using Open MPI software version 4.1.5 [1]. The exchange-correlation hybrid B3LYP functional and the valence double-zeta basis set def2-SVP were used for all atoms. UV-Vis spectra were calculated using time-dependent DFT, with a broadening of 5000 cm^{-1}.

S9 Fluorescence at different temperatures

Optical responses were obtained using a home-built microspectroscopy system. A white supercontinuum laser (SuperK EXTREME, NKT Photonic, 40 MHz) was used for sample excitation. The supercontinuum laser was monochromated by a spectrometer (Princeton Instruments, SP2150) to obtain a wavelength of 490 nm. The laser beam was focused on a sample through a 50x objective with a correction ring (Nikon, CFI L Plan EPI CR, NA = 0.7). The samples were placed on a stage cooled by flowing He liquid under vacuum (KONTI-Cryostat-Micro, CryoVac), and the sample temperature was monitored and controlled by a temperature controller (TIC 304-MA, CryoVac). We measured time-resolved PL (TRPL) using the TCSPC method with an avalanche photodetector (Becker & Hickl GmbH, ID-100-50-ULN).

Figure S1a shows the fluorescence spectra measured on the 2.0 % doped crystal with an excitation laser wavelength of 490 nm at different temperatures. The fluorescence emission is located approxiamtely at 2.1 eV (590 nm) which corresponds to the C_3^{em} emission. There is no clear temperature dependence except for the spectrum at 300 K whose area intensity is much reduced (see panel c). This can possibly be attributed

to a change in the sample position as the fluorescence intensity is largely position dependent, as shown in Figure S1b. Panle d and e show that the center of the emission energy and the full width at half maximum (FWHM) do not exhibit clear temperature dependences.

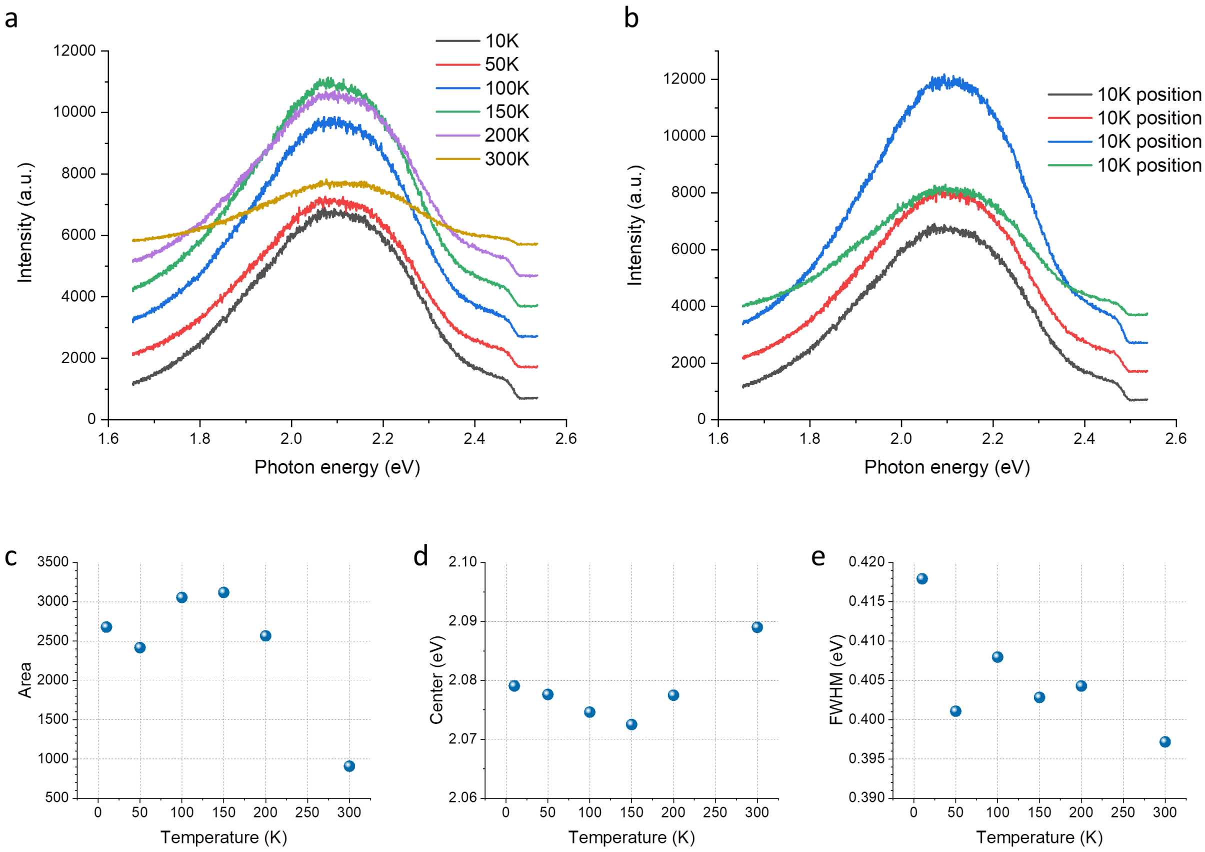

Figure S1: a) The laser fluorescence spectra measured on the 2.0 % doped crystal with an excitation laser wavelength of 490 nm at different temperatures. b) The fluorescence spectra measured at 10 K on four different spots on the crystal. The spectra are offset from one another by 1000. The temperature dependence of (c) the area intensity, (d) the energy, and (e) the full with at half maximum (FWHM) of the emission peak.

S10 Fitting analysis of fluorescence decay profiles

Figure S2 shows the fluorescence decay profiles measured at an excitation wavelength of 490 nm at different temperatures ranging from 10 to 300 K. The instrument response functions (IRF) measured at respective temperatures are plotted in blue. The decay curves are well described by a convolution of the IRF with a single-exponential decay function, $Ae^{-(t-t_0)/\tau} + \mathrm{B}$, where τ is the fluorescence lifetime, A is the amplitude, t_0 is the time shift, and B is the constant background determined from the intensity before the pulse arrival. The extracted lifetimes are 4.02, 3.88, 3.82, 3.58, 3.65, and 4.00 ns at temperatures of 10, 50, 100, 150, 200, and 300 K. The corresponding Chi-square values are 856.908, 892.175, 536.274, 736.904, 703.294 and 888.526 at these temperaturess.

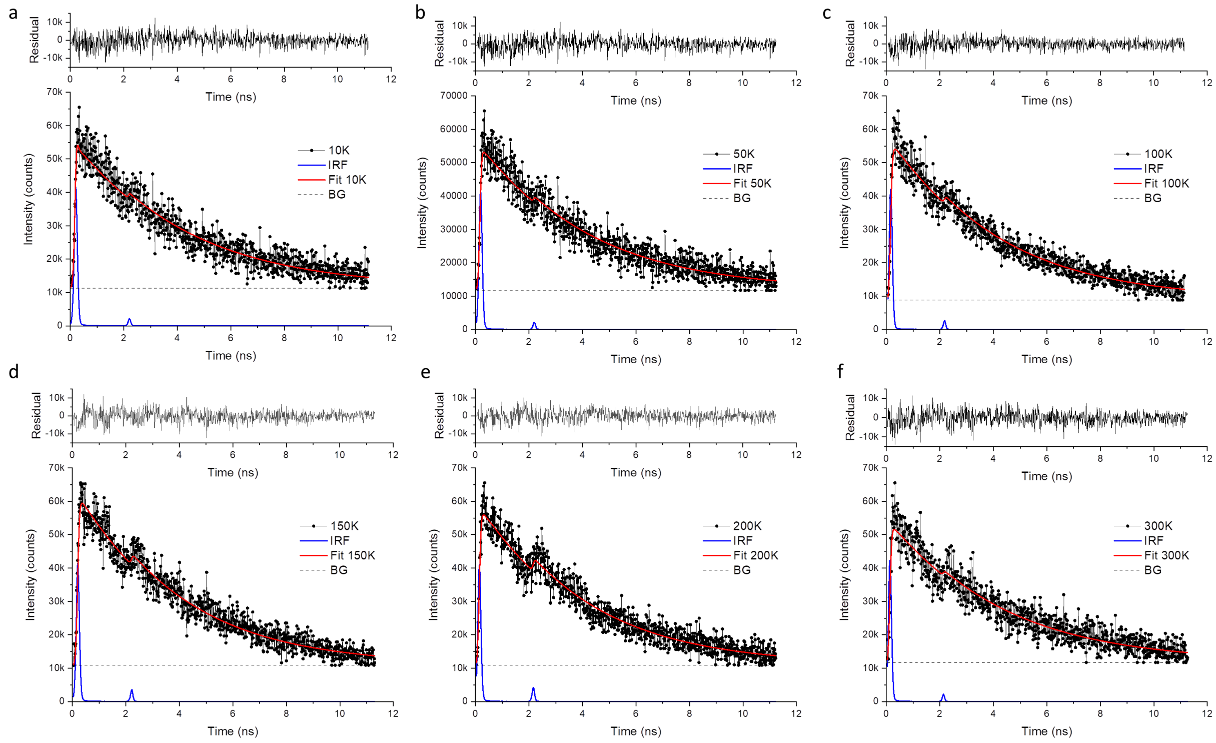

Figure S2: Fluorescence decay profiles measured at an excitation wavelength of 490 nm at different temperatures. The blue curves represent the instrument response function (IRF) at each temperature. The red curves correspond to the fitted decay profiles. The dashed horizontal lines indicate the constant background (BG) level, determined from the intensity prior to the pulse arrival. The top panels display the fit residuals.

S11 UV-Vis spectrum calculated for melamine

The UV-Vis spectrum calculated for melamine, plotted in Figure S3, exhibit no absorption bands in the visible wavelength range.

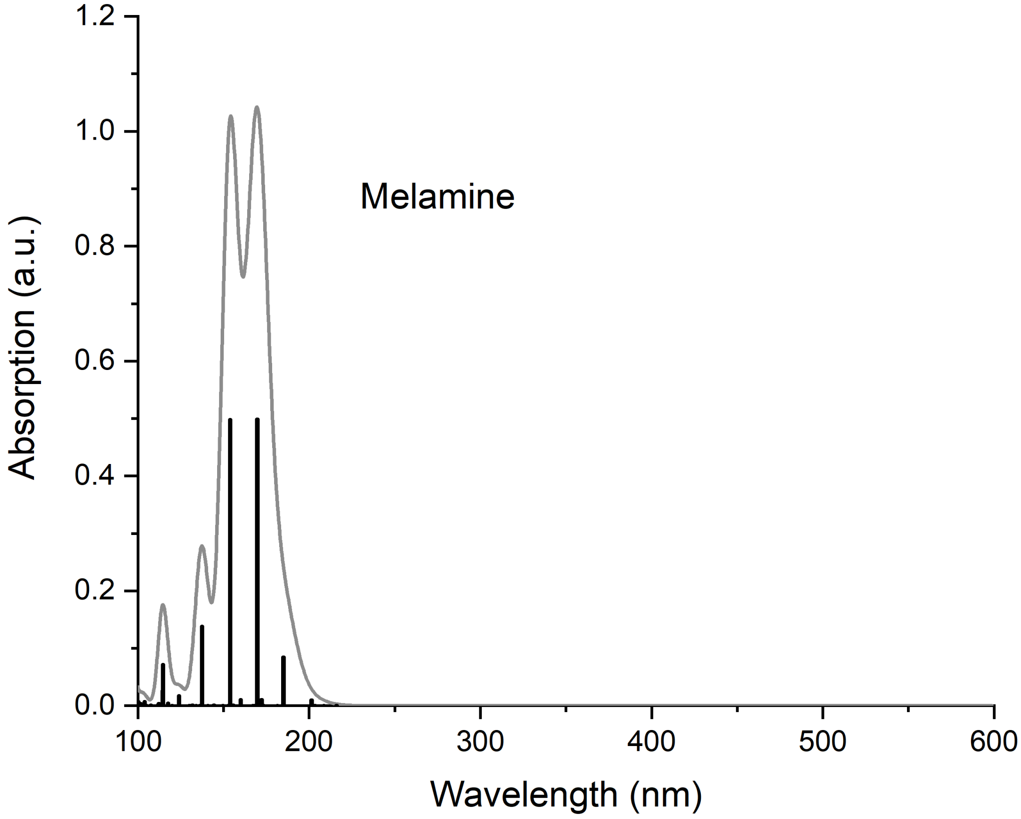

Figure S3: UV-Vis spectrum calculated for melamine in the wavelength range from 100 to 600 nm.

References

[1] Frank Neese. The orca program system. *WIREs Computational Molecular Science*, 2(1):73–78, 2012.
